# *“Research happens a lot in other settings—so why not here?”* A qualitative interview study of stakeholders’ views about advance planning for care home residents’ research participation

**DOI:** 10.1093/ageing/afae235

**Published:** 2024-10-23

**Authors:** Brittany Nocivelli, Fiona Wood, Kerenza Hood, Carolyn Wallace, Victoria Shepherd

**Affiliations:** Division of Population Medicine, School of Medicine Cardiff University, Cardiff, United Kingdom; Centre for Trials Research, School of Medicine, Cardiff University, Cardiff, United Kingdom; PRIME Centre Wales, School of Medicine, Cardiff University, Cardiff, United Kingdom; Division of Population Medicine, School of Medicine Cardiff University, Cardiff, United Kingdom; PRIME Centre Wales, School of Medicine, Cardiff University, Cardiff, United Kingdom; Centre for Trials Research, School of Medicine, Cardiff University, Cardiff, United Kingdom; PRIME Centre Wales, School of Medicine, Cardiff University, Cardiff, United Kingdom; Faculty of Life Science and Education, University of South Wales, Newport, United Kingdom; Centre for Trials Research, School of Medicine, Cardiff University, Cardiff, United Kingdom

**Keywords:** advance planning for research, qualitative research, care homes, older people

## Abstract

**Background:**

Underrepresentation of care home residents in research has resulted in a poorer evidence base for health care in care homes. Fewer opportunities to take part in research, as well as assumptions made by others about their interest or wishes, creates challenges for residents’ inclusion in research. Early discussions about research preferences and wishes may be beneficial. This qualitative study aimed to explore stakeholders’ views about how care home residents can be supported to communicate their wishes about research participation.

**Method:**

Semi-structured interviews were conducted with 25 stakeholders: care home residents (n = 5), relatives (n = 5), care home staff (n = 5), other health and social care professionals who work with care homes (n = 6), and care home researchers (n = 4). Interviews were conducted virtually or face-to-face and data were analysed using thematic analysis.

**Results:**

Views about resident research participation, the barriers and facilitators to their inclusion, and the role of advance research planning were iteratively organized into three themes: (i) We’re of no value to research; (ii) Research is difficult; and (iii) Advance research planning: good in theory, challenging in practice. Subthemes were also identified, and findings were discussed with a Patient and Public Involvement group for additional reflections.

**Conclusions:**

Stakeholders identified a number of barriers to including care home residents in research, including knowing their preferences about research. The development of interventions to facilitate communication that can be adapted to individuals’ requirements are needed to support discussions and decision-making with care home residents about wishes and preferences for future research participation.

## Key points

- The care home resident population are underrepresented in research resulting in a paucity of evidence to improve their health and social care, and quality of life.
- Stakeholders believe that care home residents are not seen as a priority by researchers and the wider community.
- Research is seen as being too difficult to get involved with for those who are less familiar with research.
- Stakeholders report challenges with relationships and the impact of communication difficulties.
- Stakeholders’ views on advance planning for research differ depending on research experience.

## Introduction

Despite their more complex health and social care needs, care home residents are underrepresented in research due to a number of barriers including practical and ethical challenges such as concerns around involving a ‘vulnerable’ population [1, 2]. Availability of good evidence to support best practice, and to improve care home residents’ quality of life, is limited as a result. Increasing the opportunities for care home residents to be involved in research is urgently needed.

Current estimates of the number of older people living in UK care homes are around 443,000 [3–6]. Given the high prevalence of cognitive impairment, co-morbidity, and polypharmacy amongst care home residents [7–9], as well as difficulties in communication for some, this population is at risk of having their health and social care needs unmet [10].

The Alzheimer’s Society estimate that 70% of care home residents have dementia [11] which, as a progressive neurodegenerative condition, is likely to affect their capacity to consent to research at some timepoint. With ethical and practical issues identified as some of the greatest challenges to conducting research in care homes internationally [1, 12, 13], facilitating early discussions with residents about their wishes and preferences about future research may be beneficial.

Advance planning procedures are available in many areas of life including the documentation of financial wishes and will writing, and Advance Care Planning (ACP) which offers individuals the chance to clarify their healthcare preferences and benefit from the autonomy this control may bring [14]. According to the Gold Standards Framework for end-of-life care [15], ACP is recognized internationally and is consistent with the Mental Capacity Act [16] in England and Wales. Despite being widely advocated for, ACP and other advance planning procedures have had limited uptake.

Advance Research Planning (ARP) has been suggested as a process to honor the research wishes and preferences of individuals who may lose capacity in relation to their inclusion in research [17]. Using elements of ARP, it may be useful to facilitate early discussions about future research preferences with care home residents, who are often considered difficult to recruit.

The views of different populations have been explored about the role that ARP activities may play in supporting preference-based inclusion in research, lending strong support for ARP as a mechanism for promoting autonomy [18, 19]. Research is needed to understand stakeholders’ views about how researchers may feasibly integrate the ARP processes into care home settings. Understanding how care home residents can be best supported to communicate their wishes and preferences could lead to the development of interventions to support care home residents to engage in important discussions about research including preferences about participating in different types of studies, or particular research activities such as routinely collected data.

This qualitative study aimed to explore the views of stakeholders (care home residents, relatives, care home staff, other health and social care professionals (HSCP), and researchers) about care home residents’ opportunities to participate in research and how best to encourage early discussions about residents’ future research participation wishes and preferences.

## Methods

### Design

Semi-structured interviews were conducted with stakeholders, either virtually or face-to-face, to enable in-depth discussions with lead researcher BN [20]. The research team have combined experience of working in care homes and close family members being care home residents. These experiences have informed study design and analysis.

### Recruitment and data collection

Participants were recruited through various routes, including contacting stakeholders who participated in a previous survey and expressed an interest in being re-contacted. Local care homes were contacted directly via email and phone calls and followed up with in-person visits. See Appendix 1 for the full details of recruitment.

A pilot interview was conducted with a participant representing a number of the stakeholder groups (family member, HSCP, and researcher) to test the interview acceptability, comprehensibility, and interview guide content (see Appendix 2). Small amendments to the design and content were made following the pilot.

This study received a favorable opinion following review by Cardiff University’s School of Medicine Research Ethics Committee (SMREC ref. 23/29). See Appendix 1 for the full details of ethical considerations.

Care home residents were supported to participate through the provision of accessible information about the interview. Only participants with capacity to consent participated in interviews as the study focused on views about research in the event of future loss of capacity. Data collection was carried out between May and September 2023.

### Data analysis

Interviews were audio recorded and transcribed. Transcripts were then checked for accuracy against the recordings and data cleaning and anonymization were undertaken. Data were analysed following Braun and Clarke’s [21] reflexive thematic analysis approach. The process of data familiarization, coding, development of initial themes, and refinement of themes was followed, supported by NVivo qualitative data analysis software (NVivo 1.7.1, QRS International). Further details about the analysis process can be found in Appendix 1.

Results have been reported using the COREQ checklist [22] (Appendix 3).

### Patient and Public Involvement consultation

This qualitative study is part of a wider study, the ENGAGE study, which has a patient and public involvement (PPI) group comprising of a relative, a member of care home staff, a relative who also has research experience, and a care home resident. This PPI group have supported the project since it commenced in January 2022, with the addition our first care home resident who joined in March 2024 during the interview stage.

A consultation meeting was held virtually with the three members who have supported the study throughout. Themes were presented and discussed, followed by a one-to-one discussion with the resident at their care home to present the same information in a more accessible format.

## Results

Details of the participants (n = 25) are presented in Table 1.

**Table 1:** Participant characteristics (*n* = 25)

	No. (%)
Stakeholder^a^ Care home resident Relative Care home staff Other health and social care professional working with care homes (HSCP) Researcher	5 (20) 7 (28) 5 (20) 5 (20) 3 (12)
Location of care home Wales England Scotland	16 (64) 7 (28) 2 (8)
Research experience (taking part in or conducting care home research) Has research experience Resident Care home staff Relative HSCP Researcher	8 (32) 0 (0) 1 (4) 1 (4) 3 (12) 3 (12)

Stakeholders who identified as a relative to someone living in a care home included relative only (n = 4), relative with experience of conducting research (n = 1) and relative and HSCP (n = 1). Care home staff member roles included manager (n = 1), senior carer (n = 1), activities coordinator (n = 1), and nursing care assistant (n = 2). HSCP roles included General Practitioner (n = 3), clinical studies officer (n = 1), and clinical quality nurse (n = 1). Length of interviews ranged from 4 to 31 min.

a Stakeholders could belong to more than one group.

A number of participants were members of more than one stakeholder group, reflecting intersections between personal and professional experiences. However, during analysis, participants were grouped based on the stakeholder category they reported primarily belonging to.

Data were iteratively organized into three themes: (i) We’re of no value to research, (ii) Research is difficult, and (iii) Advance research planning: good in theory, challenging in practice. A number of subthemes were also created and included.

### We’re of no value to research

#### Care home research as a priority to researchers and the community

Stakeholders from all groups expressed the view that care home research, and care home residents, do not seem to be a priority for researchers or the wider community. This included the view that the research community consider residents to be less valuable participants in research, perhaps because of their age or that they have a less meaningful contribution to make.

*‘I don’t think there’s the motivation to support them in the care home, and there isn’t the dynamism, or the need – the want – to make their voices heard in that particular way.’ Care home manager, P019.*

*‘I don’t think the research community as a whole think that people living in care homes – well they don’t even think they should [be included] – they would be a valuable participant in research.’ HSCP, P012.*

An experience shared by one HSCP around recruiting residents to research suggested that recruitment of this population is difficult and that researchers don’t have the time or resources to priorities their recruitment.

*‘When I was making a priority list [for recruitment] if I wasn’t able to approach everyone, unfortunately the care home residents tended to be at the bottom of the list.’ HSCP, P007.*

Participants also emphasized that the COVID-19 pandemic highlighted the paucity of care home research. Participants from all stakeholder groups shared the view that care home research has now become more of a priority because of the pandemic.

*‘I think [research in care homes] has taken longer to come onto the radar and obviously, COVID, really changed that and brought it much more into the spotlight.’ Relative with experience of conducting research, P004.*

A number of difficulties and challenges were experienced by care homes and residents during the COVID-19 pandemic. However, this led to the identification of an urgent need to priorities care home research. Some stakeholders shared that they believed researchers and research funders are now placing greater importance on care home research and allocating more resources, meaning that more care homes are getting involved with research.

*‘The pandemic catalysed a lot of my involvement with the care home sector because we recognised the vulnerability of care home residents to COVID, and that persists.’ Relative with experience of conducting research, P001.*

*“I’ll be honest, since sort of COVID we’ve started getting involved in research.” Staff member, P002.*

The experience of COVID-19 also seemed to bring into sharp relief the previous neglect of research in the care home sector.

*‘With the explosion of COVID and everything it really opened my eyes to the struggles that they have and the fact that they get forgotten and I find that really sad.’ Researcher, P014.*

#### Residents’ perceptions of their value to research

References to residents’ feelings of disempowerment and a lack of autonomy were apparent. Residents suggested that they believed they were not worthy enough to take part in research and that they, or their views, would be of no use to researchers. One resident seemed to believe that their input would not be valuable because they were no longer typical of other members of society.

*‘I think I’m past that age.’ Resident, P016.*

*“I imagine it is because one feels that you’re no longer your usual self and what use are you to anybody else.” Resident, P016.*

Further, the influence of societal beliefs towards people with disabilities or those different to ‘the norm’ was apparent and was suggested to influence the way people perceive more vulnerable populations and their ability to contribute to research.

*‘People [automatically] think if somebody’s in a wheelchair, people speak to the person who’s pushing the wheelchair. There’s that thing of asking the professionals and relatives, rather than [the individuals].’ Researcher, P013.*

Residents’ shared experiences that they do not feel respected in the care home environment in regard to their usual care needs, let alone to take part in research or in promoting their own autonomy.

*‘I haven’t got dementia thankfully and I feel that some of the staff are inclined to treat me as though I’ve lost my marbles.’ Resident, P020.*

Some relatives also gave the impression that they themselves view their relative as no longer having the ability or means to take part in research in a meaningful way. Relatives’ poor confidence in residents’ ability to participate in research was apparent and from this a resulting unwillingness to support their inclusion.

*‘He’s got dementia so I don’t understand how much he would be able to give a useful answer.’ Relative, P024.*

### Research is difficult

Some residents, relatives, and staff shared a perception that research is difficult and not for everyone. There was a general desire for more opportunities to be shared, and willingness to take part in research should it be offered, but this seemed to be hindered by the perception that research is difficult.

*‘Yeah [could participate], if it was easy.’ Resident, P021.*

Further, there was consistent reporting of a lack of awareness of care home research opportunities for care home residents, relatives, and staff members to take part in research, as well as the idea that these groups feel remote from those who conduct research, and from science.

*‘Absolutely not, no. In fact I would have said that it [research] was an almost completely alien idea to the care home staff.’ Relative, P010.*

#### Communication and relationships

Closely linked to stakeholders’ understanding and beliefs about who research is for, and what research participation entails, was the overarching notion that communication between stakeholders can be poor. Ways to improve the sharing of opportunities were suggested during interviews, including the importance of tailoring communication to ensure that it is meaningful to each potential participant.

*“It’s not easy when you’re trying to meet lots of people’s needs because you don’t want to dumb it down. Our residents a lot of them have been doctors, teachers, they’ve been quite high professionals, and still have that level of understanding but then others don’t or because of difficulties they are slower to process the information.” Staff member, P002.*

Around half of stakeholders, including residents, relatives, and staff members reported never having been approached to take part in research.

Suggestions made for improving communication and relationships included providing opportunities for residents and relatives to discuss research opportunities. Stakeholders also suggested that being involved in research, in any form, can ignite an interest to get involved. Interviewees suggested that studies would also benefit from more flexible approaches to recruitment and the formats in which research opportunities are presented to potential participants.

*‘It takes ground working, relationship building, most importantly I would say.’ Relative, P010.*

*‘I don’t think [researching in care homes] is the most natural thing to do but then I think it only takes you to get involved with one to understand that it could be made much easier for them [care home residents] and for the researchers if there was a better understanding of what was needed.’ HSCP, P009.*

## Advance research planning: good in theory, challenging in practice

### Positive views of ARP

#### Importance of ARP and the potential benefits for all

Generally, stakeholders who reported having no experience of research had a positive view about enabling ARP, as well as the value of researching the underrepresented care home population.

*‘I’ve got a firm belief that people who have dementia should and could still be involved in research. Just because somebody has lost capacity doesn’t mean it’s all or nothing, they can still decide to be involved in research, should they wish to be, just because it’s difficult we shouldn’t not do it.’ HSCP, P012.*

Further, stakeholders from all groups highlighted the importance of residents being able to share and document their wishes for future research participation should they lose capacity to make such a decision in the future. Stakeholders acknowledged the importance of becoming aware of residents’ research wishes and preferences and the possibility of implementing these in the future, should they need to. The potential benefits of ARP for residents seemed to be particularly important to relatives and staff.

*‘That’s a good idea because people’s capacity can vary, as time progresses, we can keep on fulfilling that wish. We know that this was once important to them.’ Staff member, P019.*

Further, the benefits of ARP, including improving the ability of researchers to identify and recruit participants, was discussed by stakeholders with experience of recruiting care home residents and conducting research.

Another potential benefit of improving recruitment of residents through implementing ARP was offered by a relative:

*‘There’s everything to gain to get it right, and whatever the average expectancy of a care home resident would be, that’s how many extra years that country gains of potentially enrolling that person.’ Relative, P001.*

#### Recommendations for successful implementation

Stakeholders suggested how these conversations could be successful and feasible, but primarily the focus was on how, when, and by whom the question should be raised. A number of residents considered that discussions about research participation would be appropriate during conversations about other preferences, such as their care needs, upon entering the care home. There was some agreement with this from other stakeholder groups.

*‘It could be built into their care plan.’ Staff member, P019.*

*‘It makes sense to have a conversation like we do, you know, with advance care planning, things like their wishes, do not attempt resuscitation orders, etc. Could this be added as a potential extra thought?’ HSCP, P009.*

*‘It would be nice, if it was part of the whole admission process, that you talked about future wishes for your health, future wishes for your data, future wishes for taking part in studies.’ Researcher, P008.*

There was a common view amongst stakeholder groups that family members should be involved in such discussions with residents, but for different reasons. Residents generally felt that having these conversations with family members or someone who was able to give them all of the necessary information would be best. Relatives’ answers varied with some thinking that residents needed family members present to ensure that information is shared in a way their relative can understand, but also expecting researchers to be present in order for questions to be answered. Care home staff mostly stated that family members should be included, with one staff member stating that it should be *‘somebody they trust at the end of the day’,* and that this may not be a relative for everyone. HSCP’s and researchers’ answers varied also, with some suggesting that these conversations should include whoever is most important to the resident, and others emphasizing the importance of researchers and/or senior care home staff being present and involved.

*‘I suppose somebody who is able to talk to lots of different people and are able to have an approach which can appeal.’ Resident, P016.*

*‘My daughter I would say.’ Resident, P021.*

*‘The relatives definitely … because they are more comfortable. They can explain everything, they are family so they know exactly how to explain, and if they actually do want to consent or not.’ Relative, P006.*

### Challenges of ARP

#### Concerns about capacity and residents’ ability to engage in ARP

Comments from non-resident groups suggested that relatives may underestimate the capabilities of residents.

*‘I don’t have a lot of confidence that [residents] will have the brain space to be considering it.’ Relative, P024.*

Researchers discussed the likelihood of residents’ cognitive abilities changing over time. A relative contributed a similar thought relating to concerns about changes in residents’ abilities and needs at a later time. This raises the concern that any decision made early on may no longer be reflective of the resident’s wishes at a later timepoint.

*‘[A resident doesn’t] necessarily know which kind of cognitive function [they] might have, so [they] might consent to something and then by the time it actually comes round to it, it’s very distressing.’ Relative, P023.*

Other stakeholder groups made comments related to residents’ ability to make decisions about their own research participation and understanding of consent.

*‘Some [residents] won’t understand the concept of capacity.’ Researcher, P008.*

Staff members suggested that being involved in facilitating discussions about ARP may be difficult due to work pressures, linking with other comments identifying care home staff as possible barriers to the inclusion of residents in research.

Further, those with experience of conducting research were more wary or hesitant about ARP in terms of its implementation feasibility.

*‘It’s quite difficult to do. I mean, I like it in theory … I don’t think it would work in practice.’ Researcher, P008.*

Further, a focus was placed on the potentially sensitive nature of advance consent discussions. A number of respondents commented on the distress that having conversations surrounding potential future incapacity may cause to care home residents, especially considering the hypothetical nature of the topic.

*‘I think the idea of talking to somebody about an uncertain future, and what they might want to do, when things are potentially worse than they are now, it’s quite ethically complicated, in terms of how distressing it might be to imagine that uncertain future.’ Relative, P004.*

However, this view was not shared by residents themselves who thought that residents would be happy to have these conversations.

#### Binding element of ARP

One main contested point was whether ARP conversations would provide a binding contract from which residents, as a ‘vulnerable’ population, may be taken advantage of at a later date. However, mention of it being ‘hard’ to get consent for residents may reflect concern for the resident’s wellbeing or highlight the likelihood of it requiring more effort from stakeholders in supporting residents.

*‘There is a difficulty because [residents] are perhaps vulnerable, and getting consent for vulnerable people is hard.’ Relative., P024.*

Further, an underlying belief that an advance discussion would commit them to a prior decision, over and above their preference and needs at that time was apparent.

*‘I don’t think you can get a blanket agreement to participate in research for all kinds, I think that wouldn’t be ethical to consent [in advance].’ Relative, P010.*

Many stakeholders suggested that a general agreement to participate can be ethically obtained from residents during an early discussion about wishes and preferences, but that this needs to be revised each time a new research opportunity arises in order to assess the abilities and suitability for the resident at that time.

#### Implementation challenges

Participants identified potential challenges to the implementation of ARP, including concerns over the feasibility of having these discussions. Factors such as access to care homes and residents at an appropriate time, the language used, and turnover of residents, may pose barriers to facilitating discussions about ARP.

*‘It’s not like you could just turn up to a care home every week and try and speak to anyone who’s entered newly.’ HSCP, P009.*

*‘Having questions that are inclusive of the likely eventualities like losing capacity and what that means in lay terms, [for example] my nana would not know what capacity means even if you explained it to her a thousand and one times.’ Relative, P001.*

Further, relating to issues around communication and relationships previously mentioned, challenges were identified around who the ‘right person’ to ask such questions, or facilitate such discussions, would be. Trust in the person asking questions and facilitating a potentially distressing topic of conversation seemed to be another important factor for one relative.

*‘It’s not just about content, it’s also about the execution, the format of delivery and who’s the right person, you run the risk then of there just being a check box exercise where people are talking about it half-baked.’ Relative, P001.*

### PPI Consultation

Overall, discussions held with PPI members supported, and strengthened, our initial theme development. Members shared important views and experiences related to the themes and supporting quotes. Table 2 summarizes the main discussion points and resulting changes to our findings.

**Table 2:** Summary of PPI consultations

Theme	Discussion points	Changes made as a result
We’re of no value to research	PPI members were disheartened about this theme but there was a shared understanding about why this came up throughout interviews. Experiences were shared of relatives overriding residents’ decisions regardless of resident having capacity. Resident PPI member shared that her first opportunity to take part in research was following COVID-19, in a pandemic-related study.	We carefully considered the sensitivity of this identified theme and how we discussed it in the paper. Such experiences provided evidence which helped strengthen our explanations as to why residents may feel disempowered or have a perceived lack of autonomy. This evidence supported suggestions made by stakeholders that COVID-19 has changed, and improved, priority of care home research that we made sure to emphasize.
Research is difficult	Research concepts need more explanation and people need to be educated about what it really is and what participation entails. A PPI member with experience as both a relative and researcher shared the benefit of the relationship that they have been able to build with staff in terms of sharing research opportunities and their reception.	More of a focus was placed on suggestions that, in any future intervention, educating stakeholders about research (types of design, process etc.) and making it easier to get involved, is essential. This view strengthened our suggestion that a focus on improving communication and relationships between stakeholders can facilitate positive changes for research participation.
Advance Research Planning – Good in theory, challenging in practice	This theme identified from the data was expected by PPI members due to reported experiences of general difficulties in communication in care homes between stakeholders (including tailoring communication to residents and understanding exactly what it is residents want). A resident PPI member shared her willingness to help with research and the importance she places on contributing to society.	More support is needed to promote communication of ARP. The suggestion that residents are willing to take part in research but are limited due to lack of opportunities and other barriers to their inclusion, was strengthened and more emphasis was placed on the suggestion that research needs to be easier to access and tailored to potential participants’ needs.
Future steps	*‘The communication needs to come first’.* A resident PPI member discussed how much looking at photographs meant to her and that photographs would be beneficial to residents. A PPI member also shared that group discussions or talks by researchers may prevent residents from asking questions.	This comment helped emphasize the importance of, and suggestion that, communication is one of the greatest barriers that could be targeted following this research. Sharing of preferences helped us to think of potential resources for future intervention development. We have thought more about successful ways to facilitate a future intervention. For example, it seems that the intervention should target residents and their relatives individually, rather than within a group setting.

## Discussion

Facilitating early discussions with care home residents about their research participation wishes and preferences has the potential to benefit research participation, and thus representation, of a population with complex health and social care needs at risk of their needs being left unmet. There is an apparent need for more research in care homes, not only for research specific to care home residents but also studies for which residents meet inclusion criteria.

Stakeholders discussed the importance of research including the care home population but also the challenges of doing so, in line with previous studies [1, 12]. With regard to the apparent importance, yet low priority status, of care home research, stakeholders discussed the impact of the COVID-19 pandemic. Their perception that care home research has become more of a priority post pandemic aligns with recognition of improving research in care homes by government bodies. For example, following the pandemic, an announcement was made by the UK Health Security Agency of a data sharing scheme for over 500 care homes in England to monitor infections in care homes [23, 24].

Furthermore, recruitment experiences of stakeholders, suggest that in an attempt to be efficient with resources researchers opt to recruit less ‘hard to reach’ populations. It is important to recognize that research participation, whilst often viewed as burdensome, is a right which should not be denied due to residence in a care home. Rights-based approaches are being established to promote research inclusivity people living with dementia in countries such as Canada (e.g. [25]). Furthermore, the resulting lack of inclusion may facilitate residents’ feelings that they are less valuable to research or to society in general, identified in this study, which has also been reported in other studies [1]. Awareness of this may be especially difficult for a generation considered to place importance on contributing to society [26]. Additional findings of resident disempowerment and lack of perceived autonomy in this study align with previous care home research which reports that, in some cases, experiencing a lack of support to make their own decisions can result in residents giving up trying to express their voices and agency [27].

It is possible that society instils the narrative that when people get older, or in any way impaired, they are automatically less able to take part in activities they once could. Experiences of being overruled, despite knowing they are capable, may lead to residents feeling that they are no longer in control of their own lives. The resulting apparent lack of interest in getting involved in research has been reported in a review of barriers and facilitators to the inclusion of this population in research [1], and may be due to the feeling that events are out of their control (explained by theories such as learned helplessness, [28]). It is possible that the implementation of advance planning for research in care homes may increase residents’ perceived control over their lives and thus improve feelings of autonomy too, in line with principles of Self-Determination Theory [29]. Further, such feelings may be due to prejudiced views about this population, as reflected in the ‘Research is difficult’ theme, and the wariness and hostility towards researchers sometimes seen, as suggested by relative PPI member. These findings strengthen other research findings that residents share feelings of not belonging and that poor relationships are factors which consequently facilitate social loneliness for care home residents [30]. Fricker’s [31] philosophical framework of epistemic injustice lends support to this, explaining that epistemic injustice manifests as an exclusion of marginalized and oppressed people from being heard and understood by others, as seen with the care home resident population and their under-representation in research.

Whilst this is the first study to explore advance planning for research in the care home population, the wider literature looking at translating advance planning for research participation into practice also discusses optimum conditions in which stakeholders believe implementation would be most successful. In a survey of public and professional stakeholders, Shepherd et al. [19] shared that participants discussed the importance of with whom, and when, discussions would be most successful, as well as other crucial contextual and resource requirements that would be optimal to implement advance planning discussions in an acceptable and feasible manner. Further, stakeholders of a workshop held by Ries et al. [17], focusing on research involving people with dementia, included suggestions of utilizing a ‘phased approach’, and also the importance of raising awareness of advance planning for research, in line with the findings of the present study.

In addition, it is important to consider that advance planning for research participation would need to operate within the UK policy framework governing health and social care research (i.e. the Health Research Authority [32]), considering recommendations within proposed principles including scientific and ethical conduct, safety, and benefits and risks.

Furthermore, our findings are consistent with reviews of care home research which report that communication and relationships often act as barriers to the inclusion of care home residents in research [1, 12]. Previous research findings have suggested that superficial relationships are often seen between stakeholders in care home research [30].

### Strengths and limitations

This qualitative interview study used a reflexive thematic analysis approach with iterative data collection and analysis enabling a richer and broader understanding of participants’ views and experiences. The modest sample size meant that participants may not reflect a broad range of perspectives including people from different socio-economic, educational, ethnic, and geographical backgrounds. This has also meant we were not able to compare and contrast views from different groups of people. However, we achieved good representation of stakeholders supplemented by additional input from our PPI group.

We recognize the difficulty we experienced in capturing the voices of care home residents with research experience. We faced challenges recruiting residents with research experience as they were cautious about the formal consent process despite conversing happily and freely ‘off the record’ before consent was formally audio recorded, which has been reported in other studies [33]. In order to include and supplement the views of residents, we recruited a resident with experience of research as new member of the PPI group.

### Future research

Understanding who stakeholders believe would be best to support revision of consent, including revision based on the type of research opportunity at that time or interpretation of previous expressed preferences in relation to present needs, would be a useful next step for researchers to compliment this research area. Further research is also needed to explore the views of regulators such as ethics committees.

General education about research, as well as the aims of advance planning discussions would need to preface early discussions, with communication being key. Information shared with stakeholders should be tailored and accessible to each stakeholder group and be a supportive tool to help individuals understand all of their options, and make informed decisions based on these. In line with our study findings, recommendations to enhance opportunities for residents to express their wishes and preferences for research can be found in Table 3.

**Table 3:** Recommendations to enhance opportunities for residents to express their research participation wishes and preferences

Recommendations by stakeholder group
Residents 1. Residents may benefit from having greater awareness about research generally in order to maximize their understanding about what participation entails. 2. Residents should be supported to express their wishes and preferences in advance, but also at the time of any proposed research, considering that these may change with time.
Relatives 3. Relatives may benefit from greater awareness about research generally in order to maximize their understanding about what participation entails. 4. Relatives should support residents to share their own views, wishes, and preferences about taking part in a study rather than making assumptions based on their own views. 5. If consenting on a resident’s behalf, relatives should base their decision on the resident’s wishes and preferences rather than their own. 6. Relatives should be supported to engage in processes which allow residents to share their own views, wishes, and preferences.
Care home staff 7. Care home staff may benefit from greater awareness about research generally in order to maximize their understanding about what participation entails 8. Care home staff play an important role in the sharing of research opportunities, recruitment of residents, and retention of residents in research – staff can help to bridge the gap between researchers, residents, and relatives by helping to share positive messages about research.
Other Health and Social Care Professionals 9. Health and Social Care Professionals who are involved with care homes can help raise awareness of opportunities for care home residents to be involved in research projects.
Researchers 10. Researchers should consider how to ensure that residents have an equitable ability to participate in research which should not be denied due to residence in a care home. 11. Researchers should consider how to include care home residents in a broader range of studies not only for research studies specific to care homes. 12. Researchers should consider how to maximize opportunities for residents to express their views about research. 13. Researchers should ensure that discussions about research are relevant, appropriate, flexible and tailored to the resident’s communication needs.
Regulators 14. Regulators should consider the importance of including care home residents in research and allow for reasonable adjustments to be made to support their inclusion. Future work should focus on identifying and addressing the regulatory barriers to inclusion.

We are currently developing a communication intervention to support residents to engage in early discussions about their research wishes and preferences.

## Conclusions

Stakeholders in this study express the importance of care home resident representation in research but also recognize a number of barriers to their inclusion including communication difficulties. Due to the greater likelihood of residents losing capacity to consent at a future timepoint, early discussions about wishes and preferences could benefit not only them, but their potential future consultees, researchers, and the generalizability of health care research findings to the wider care home population. Internationally, planning ahead for research with other ‘vulnerable’ populations is of huge importance. Facilitating such discussions with care home residents is a complex process and requires support from other individuals who hold their trust. The findings of this study can contribute to the development of an adaptable communication tool needed to support discussions and decision-making with care home residents about their wishes and preferences for future research participation.

### Regulators

Regulators should consider the importance of including care home residents in research and allow for reasonable adjustments to be made to support their inclusion. Future work should focus on identifying and addressing the regulatory barriers to inclusion.

## Supplementary Material

aa-24-0996-File002_afae235

## Data Availability

The dataset generated and used in this study is available through submission of a data request to the Centre for Trials Research at https://www.cardiff.ac.uk/centre-for-trials-research/about-us/data-requests.
