# Supplementary material for: “Research happens a lot in other settings—so why not here?” A qualitative interview study of stakeholders’ views about advance planning for care home residents’ research participation
Source: Age Ageing. 2024 Oct 23;53(10):afae235. doi: 10.1093/ageing/afae235 (PMC11499620; doi:10.1093/ageing/afae235)
Supplement: aa-24-0996-File002_afae235 [file aa-24-0996-file002_afae235.docx]

**“Research happens a lot in other settings – so why not here?” A qualitative interview study of stakeholders’ views about advance planning for care home residents’ research participation. (AA-24-0996.R1)**

**Appendix 1. Further details of methods**

*Recruitment*

Researchers of on-going care home studies, found via the National Institute of Health Research website (<https://fundingawards.nihr.ac.uk>), were contacted via email. Existing networks, such as Enabling Research in Care Homes (ENRICH, ENRICH Cymru and ENRICH Scotland) and social media (Facebook and Twitter) were utilised to recruit participants, and opportunistic recruitment of researchers was also conducted at relevant conferences.

*Ethical considerations*

Participants received an information sheet and consent form prior to the interview as well as the opportunity to ask questions. Participants provided verbal consent before taking part in the interview, which was audio recorded in accordance with guidance provided by the Health Research Authority (HRA; [21]). Unique study ID numbers were allocated to participants to ensure anonymity, as well as the removal of any identifying information from transcripts prior to analysis.

*Data analysis*

The lead author undertook analysis of the first 10 transcripts, identifying codes and developing initial themes, with discussion with the wider research team. Following this, a random selection (n=5) of transcripts were sent to the wider team for review along with an early coding framework. The team then met to discuss and refine themes. During analysis the team decided to probe more specifically into some topics and to focus recruitment efforts on under-represented stakeholder groups. The remaining transcripts were coded by the lead researcher and further development and refinement of themes was carried out iteratively through ongoing discussions with the wider research team.

**Appendix 2. Interview guide**

THANK YOU FOR PARTICIPATING IN OUR RESEARCH

YOU WILL BE OFFERED A COPY OF THIS CONSENT FORM TO KEEP


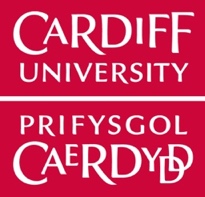


**ENGAGE Study Interview Topic Guide**

**Introductions**

- Introductions - thank you very much for taking part.
- Introduce myself – my PhD project focuses on improving engagement of care home residents in research with the goal of developing an intervention to help support the inclusion of residents in research
- Check receipt and understanding of the PIS
- The main aim of this interview is to get your views about care home residents participating in research and early discussions about participation wishes.
- What we talk about today will be kept confidential. We might use some quotes from what you’ve told us in presentations and publications, but your name (and anyone else’s name) will not be used.
- If there are any questions you don’t want to answer, or if you would like to stop the conversation or recording at any time, please let me know.
- I may also make some notes during the interview to add more information to what is being said.
- Do you have any questions?
- If you don’t have any other questions, I’ll start by reading the statements on the consent form and asking you to verbally confirm that you agree with each one.

> Start audio-recording

> Obtain consent

> Stop recording and start new audio-recording

**Participants ‘About You’**

- Would you class yourself as a care home resident, staff member, relative, researcher, or other health and care professional who works with care homes?
- How long have you been working/living in X?
- Area you live/work in? [local authority level area]

**Residents participating in research**

Resident/relative/staff member/health care professional (HCP)

- Do you have any experience with research? What is your understanding of what research is?
- Could you tell me about any opportunities you/your relative have had to take part in research studies in the care home?

Prompt

- How often do you hear about opportunities to get involved with research?
- Who shares opportunities about participating in research with you?
- Would you like more opportunities to get involved in research?

Researcher/HCP

- Could you tell me about how you recruit care home residents to your research studies?

Prompt

- How often do you share opportunities for residents to get involved with research?
- Who shares these opportunities about participating in research?

**Barriers and facilitators to residents participating in research**

- Why do you think care home residents participate in research studies less than other people might do?

Prompt

- Do you think anything prevents opportunities from reaching residents? Do you think researchers are able to design their research to be fully inclusive?
- What do you think would increase research participation/inclusion?

**Early discussions about residents wishes and preferences for future research participation/advance planning for research**

- Some people have suggested that it might be a good idea to have early discussions with residents about their wishes and preferences for their own research participation. What do you think about that?
- When do you think would be the best time to have these conversations?
- Who should the resident be having these discussions with? [Care home manager, named care worker?]
- Do you think that residents would be happy to answer questions about their future preferences should they lose capacity?

Prompts

- For example, should you/a resident lose capacity to consent to taking part in research in the future, then do you think their previous wishes about participating should be considered?

**End of Interview**

- All of the questions I wanted to ask have been covered – before we finish is there anything I haven’t covered that you would like to discuss?
- Thank you for taking the time to speak with me and share your views
- Stop audio recording

**Debrief**

- Thank participant for taking part in the interview.
- Reminder that we will keep all the information confidential.
- Do you have any further questions?
- I will be in touch once all the information has been analysed to send you a summary.
- You have my contact details - if you would like to get in touch in the meantime, feel free to contact me
- Provide vouchers

**
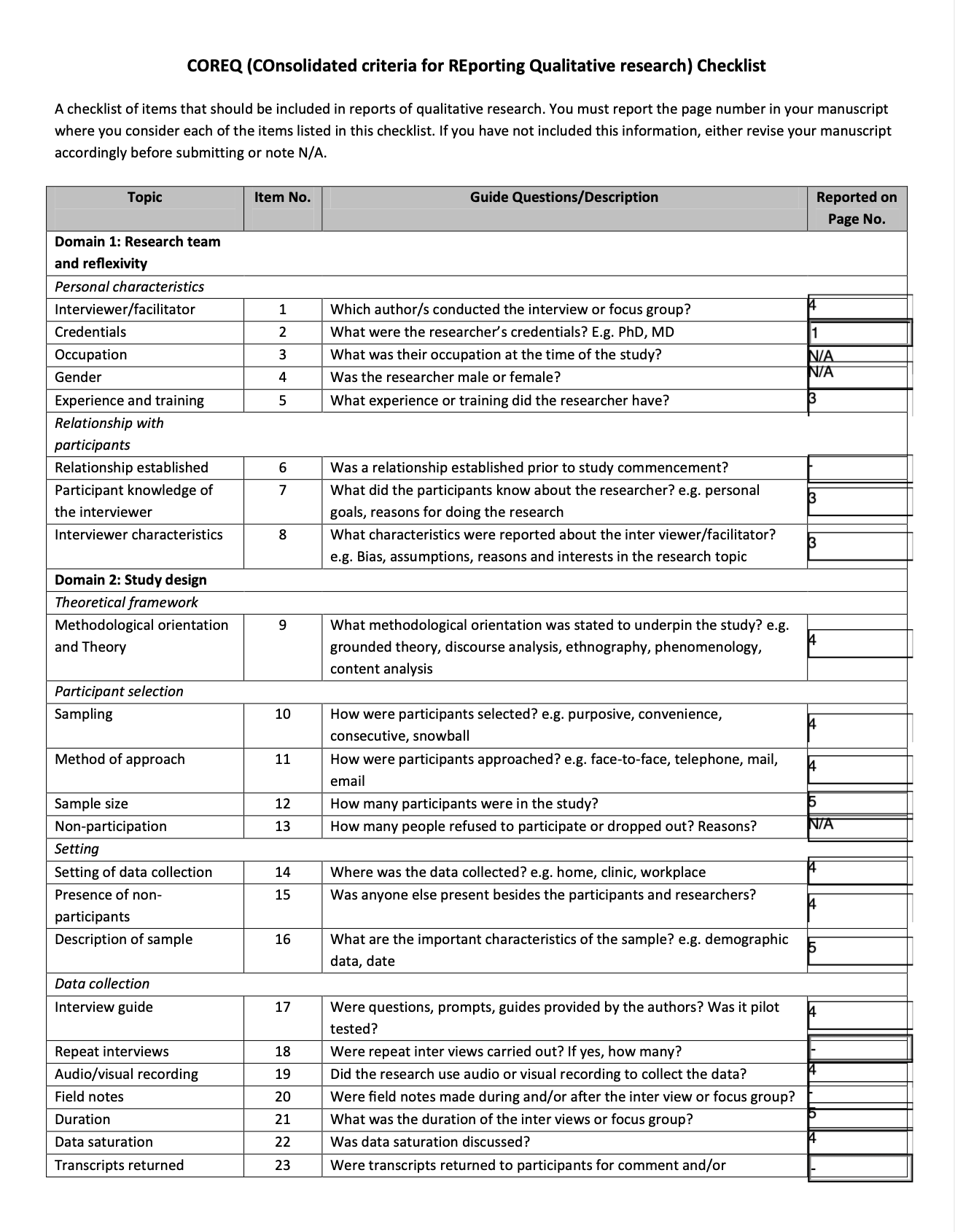
Appendix 3. COREQ checklist**

**
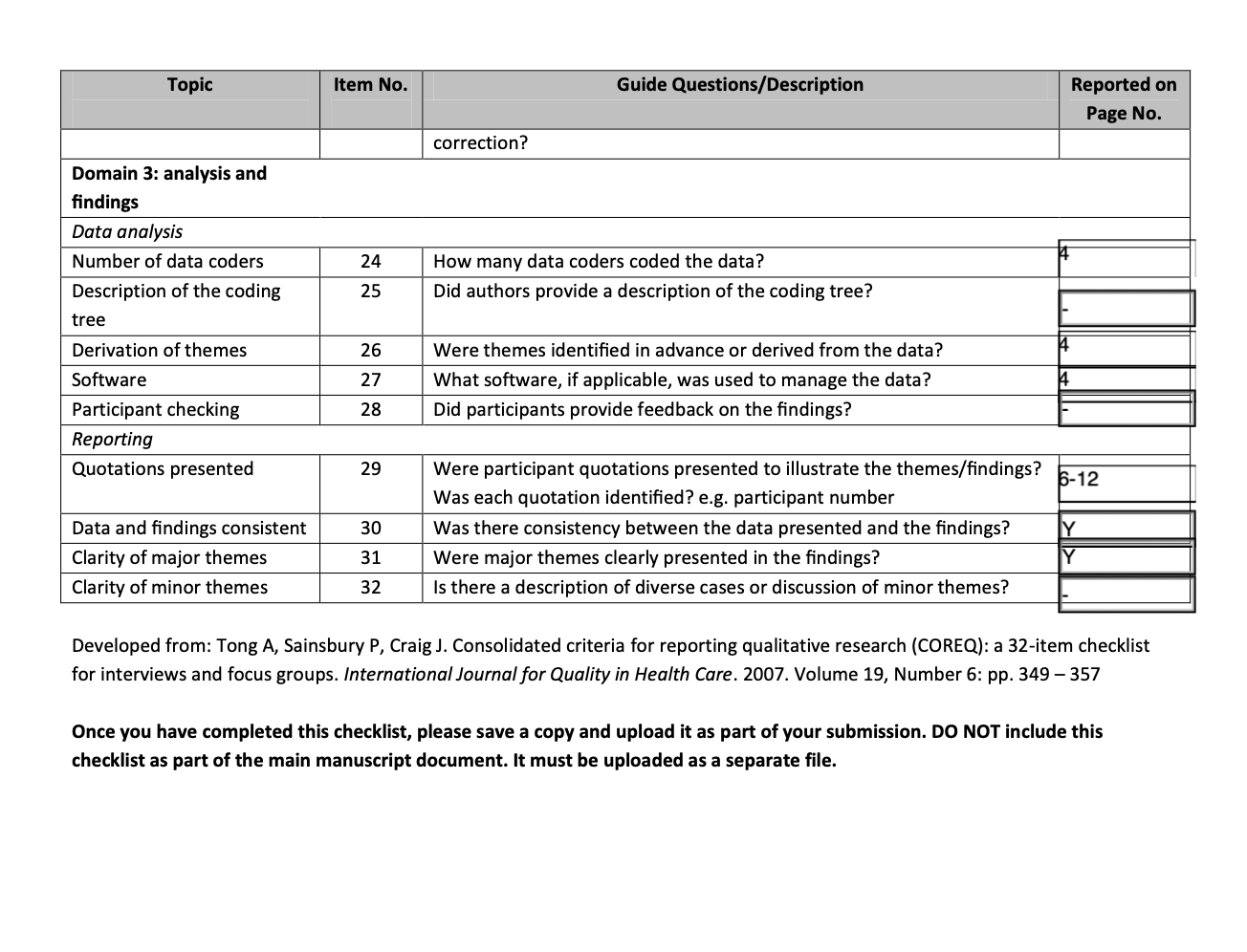
**
